# The impact of the Scandinavian Neurotrauma Committee guidelines for pediatric head trauma on the management in the emergency department—a retrospective time series analysis

**DOI:** 10.1186/s13049-026-01554-z

**Published:** 2026-01-19

**Authors:** Johan Allgoth, Johanna Berg, Jens Wretborn

**Affiliations:** 1 Department of Emergency and Internal Medicine, Skane University Hospital, Malmo, Sweden; 2 Department of Global Public Health, Karolinska Institutet, Stockholm, Sweden; 3 Department of Clinical Sciences, Lund University, Lund, Sweden; 4 Clinical Department of Emergency Medicine, Region Östergötland, Linköping, Sweden; 5 Department of Biomedical and Clinical Sciences, Linköping University, Linköping, Sweden

**Keywords:** Head trauma, Guidelines, Pediatric, Emergency department

## Abstract

**Background:**

The Scandinavian Neurotrauma Committees (SNC) guideline is a validated and well established decision tool for pediatric minor to moderate head trauma in Sweden, categorizing patients into low, moderate and high risk for intracranial injury. It recommends observation over imaging with computer tomography in low and moderate risk stratified patients. However the effect on guideline implementation on emergency department length of stay and imaging rates with computer tomography is unknown.

**Objective:**

Investigate the effects of the implementation of the SNC guidelines for pediatric minor head injury on utilization of computer tomography, emergency department length of stay and inhospital admission.

**Methods:**

A retrospective observation study between January 2011 and December 2022 in a health care system in south east Sweden where SNCs guideline was implemented at the beginning of 2017. Computer tomography imaging rates, emergency department length of stay and hospital admission was compared for pediatric visits with a chief complaint of head injury made before the implementation of the SNCs guidelines in January 2017 was compared with visits made after the implementation using segmented time series analysis.

**Results:**

A total of 16,244 visits pre-implementation were compared with 16,164 post-implementation. Death, neurosurgical intervention and intracranial hemorrhage was rare (< 0.1% for each outcome) and did not differ between the pre-implementation and post-implementation group. Computer tomography rates were 3.3% before and 2.7% after implementation.The time-series analysis showed that the majority of the reduction happened pre-implementation (14% yearly decrease) compared to post-implementation (1.6% yearly decrease). Emergency department length of stay did not differ at 89 (interquartile range 50–150) vs 91 (interquartile range 45–159) (*p* = 0.11) minutes respectively. Hospital admissions showed a continual decrease during the whole study period with little effect of the guideline implementation (13% vs 12% yearly decrease, *p* = 0.6).

**Conclusion:**

There were lower rates of computer tomography in pediatric patients with minor to moderate head trauma after the implementation of the SNCs guidelines but the majority of the reduction in imaging happened before the guideline implementation.

**Supplementary Information:**

The online version contains supplementary material available at 10.1186/s13049-026-01554-z.

## Introduction

### Background

Head injury is a common reason for emergency department (ED) visits among children worldwide [1] with about 1 850 visits per 100 000 citizens in Sweden [2]. Head injuries are classified as minimal, mild, moderate, severe or critical [3]. Minimal and mild head injuries are usually referred to as minor head injuries and compose 80–90% of all head injuries in children [4–6]. Of the minor head injuries, 4–6% have pathological findings on initial computed tomography (CT) scan and 0.1–0.2% require neurosurgical intervention [4]. The rarity of serious adverse events poses a diagnostic challenge when the golden standard investigation (CT) comes with a long-term increased risk for radiation related cancer, particularly relevant in children [4, 5, 7].

In 2010 the Pediatric Emergency Care Applied Research Network (PECARN) presented guidelines for the initial management of children with head injury to standardise workup and reduce the number of CTs [4]. In 2016 the Scandinavian Neurotrauma Committee (SNC) presented similar guidelines for children with minor to moderate head injury, adjusted for a Scandinavian setting, based on evidence as well as consensus opinions [5]. In these guidelines head injury is categorized as moderate, mild with high/moderate/low risk or minimal. For moderate and mild injury with high risk, CT and admission for observation for 24 h are recommended. For mild injury with moderate or low risk, CT or observation for 12 or 6 h respectively are equal options. For minimal head injury no CT or observation is needed.

According to a Swedish survey study in 2022, 76% of Swedish ED:s managing children with head injuries had implemented a guideline for the initial management of children with minor head injury, of which 55% used the SNC guidelines [6]. Recently, the SNC guidelines have been prospectively validated, demonstrating the ability to identify cases with significant injury on CT or neurosurgical intervention [8]. Whether the identification comes with increased, or reduced, resource utilisation related to CT or admission is unknown. The rate of CT in the validation study of the SNC guidelines reported a 3.4% CT rate if the guidelines were applied, which is lower compared to the CT rate in North America prior to the PECARN guidelines [4, 8]. However, the base rate for CT in pediatric minor head injury is unknown [9]. Furthermore, the option within the SNC guideline to either image or observe patients deemed to have mild injury makes it difficult to estimate the impact of the guidelines on both imaging rates, admissions and ED length of stay [5].

## Methods

### Aim

The aim of this study is to investigate the effects of the implementation of the SNC guidelines for pediatric minor head injury on utilization of CT, ED length of stay and inhospital admission.

### Study design and setting

This was a retrospective observational study at three EDs in a regional healthcare system in the southeast of Sweden (Region Östergötland). These three EDs, one academic tertiary care center (Linköping University Hospital), one urban community (Vrinnevisjukhuset, Norrköping) and one rural ED (Motala Lasarett) together serve all of the 450 000 citizens. All EDs use the RETTS triage system, used by a majority of EDs in Sweden [10] which triage patients on a scale from 1 (immediate) to 5 (non-urgent) based on vital signs and chief-complaint specific considerations [11]. In January 2017, the tertiary care center formally implemented the SNC guidelines as their local guidelines for minor head injury in children. While the rural and urban ED lack local guidelines, the rural ED are staffed by physicians from the tertiary care center. The urban ED implemented similar routines based on the SNC guidelines during the end of 2016. The study was approved by the Swedish Ethical Review Authority (dnr 2022-03404-01).

### Participants

ED visits done by patients under the age of 18 with minor to moderate traumatic head injury were included in the study. Defined as an ED visit with chief complaint of trauma to the head and an initial triage acuity lower than the highest, *immediate* acuity, which corresponds well with the definitions of moderate to minor traumatic head injury in the SNC guidelines. Patients with a chief complaint of trauma were not included in the study.

### Variables

We collected data on age, mode of arrival (ambulance, walk-in or other), triage acuity (1–5) for all included patients. The primary outcome was diagnostic imaging with CT within 24 h of the ED visit. Secondary outcomes included ED length of stay, hospital admission, intracranial hemorrhage or neurosurgical intervention up to 30 days from ED visit and 1-year mortality. The exposure was an ED visit before implementation of the SNC guidelines (2011–2016) or after implementation (2017–2022).

### Data sources/measurements

Demographic data and length of stay was extracted from the electronic health record (EHR) database shared between all three study sites. Diagnostic imaging status was retrieved from imaging databases, querying for any imaging study performed within 30 days of the ED visit. Neurosurgery was defined as a registered neurosurgical ICD-codes within 30 days of the visit and covers all relevant care within the healthcare system. Death was matched with the national tax registry, through the EHR which covers all deaths of Swedish citizens.

### Study size

This was a retrospective complete cohort time series analysis where conventional sample size analysis is less suitable and none were performed prior to initiation of the study.

### Statistical methods

Descriptive data are presented as mean with SD and non-parametric data as median with interquartile range (IQR) as appropriate. We used Chi-squared (admissions), Wilcoxon rank sum (length of stay) and Fischer’s exact test (neurosurgery) for univariate analysis between groups. To model the effect of guideline implementation on the management of pediatric head injury, we used a segmented regression model of interrupted time series data with a poisson distribution. Segmented regression is considered a strong quasi-experimental statistical method for evaluating interventions and allows for both a level change (immediate shift) analysis and a slope change (trend over time) analysis following the intervention [12–14]. All visits were aggregated by quarter and the pre- and post-intervention periods defined using a binary indicator variable. Analysis was done in R (version 2024.12.0).

## Results

**Fig. 1:**
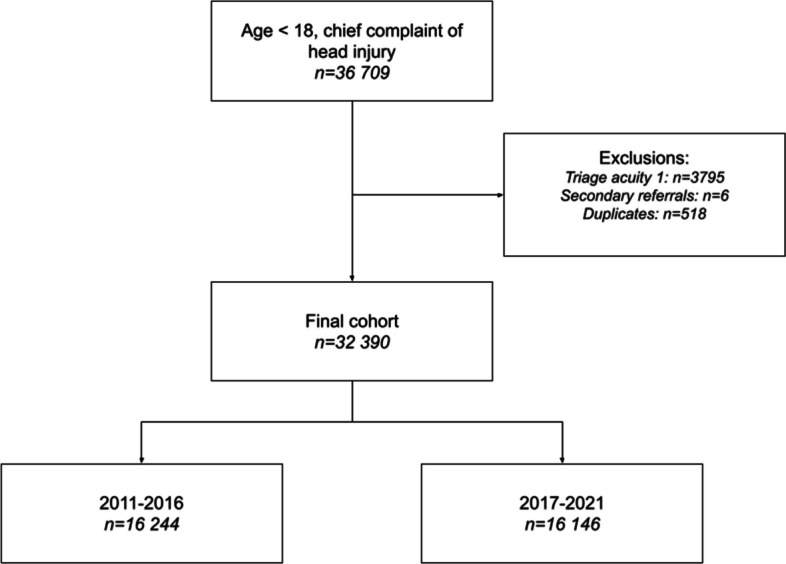
Flow-chart of patient selection

After exclusion of 4319 patients, a total 32,390 ED visits were included in the study, of which 16 146 (50%) visits were done after the implementation of the SNC guidelines (Fig. 1). There were less triage acuity 3 (urgent) and more triage acuity 4 (less urgent) and 5 (non-urgent) and fewer patients arriving by ambulance in the post-implementation period (Table 1).

**Table 1:** Demographic information of the 2011–2016 (pre-implementation) and 2017–2021 (post-implementation) groups

**Characteristic**	**2011—2016** *N* = 16,244^a^	**2017—2021** *N* = 16,146^a^
Gender		
Female	6,630 (41%)	6,326 (39%)
Age	4.0 (2.0, 10.0)	5.0 (2.0, 9.0)
Triage-level		
2 (very urgent)	1,109 (6.8%)	850 (5.3%)
3 (urgent)	6,005 (37%)	4,023 (25%)
4 (standard)	5,145 (32%)	7,066 (44%)
5 (non-urgent)	1,158 (7.1%)	2,779 (17%)
Unknown	2,827 (17%)	1,428 (8.7%)
Arrived by:		
Ambulance	1,725 (11%)	1,008 (6.2%)
Walking	13,944 (86%)	14,644 (91%)
Other	29 (0.2%)	9 (< 0.1%)
Unknown	546 (3.4%)	485 (3.0%)

^a^n (%); Median (Q1, Q3)

The number of CTs was lower in the post-implementation cohort with 437 (2.7%) compared to 528 (3.3%) pre-intervention (*p* = 0.002). ED length of stay was unchanged at 89 (IQR 50–150) vs 91 (IQR 45–159) (*p* = 0.11) minutes and serious adverse events were rare and comparable in both groups at less than 0.1% for intracranial hemorrhage, neurosurgery and death within one year respectively. There were fewer admissions during the post-implementation period at 7.4% compared to 11% pre-implementation (*p* < 0.001) (Table 2).

**Table 2:** Outcomes of the 2011–2016 (pre-implementation) and 2017–2021 (post-implementation) groups

**Characteristic**	**2011—2016** *N* = 16,055^a^	**2017—2021** *N* = 16,121^a^
Length of stay	89 (50, 150)	91 (45, 159)
Dead within 1 year	1 (< 0.1%)	3 (< 0.1%)
Computer Tomography	528 (3.3%)	437 (2.7%)
Admitted	1,775 (11%)	1,191 (7.4%)
Intracranial hemorrhage	8 (< 0.1%)	11 (< 0.1%)
Neurosurgery performed	2 (< 0.1%)	1 (< 0.1%)

^a^ n (%); Median (Q1, Q3)

**Fig. 2:**
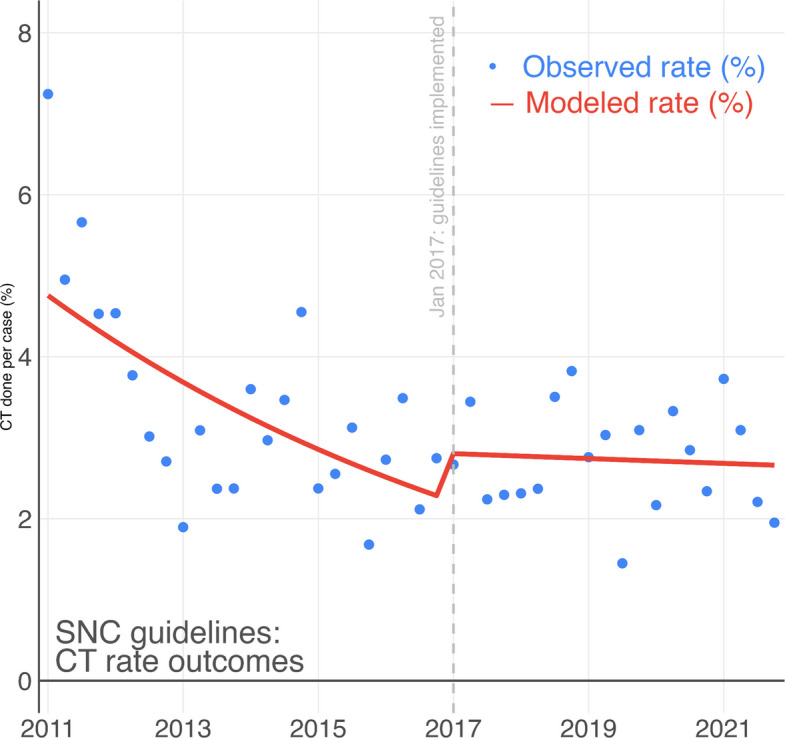
Interrupted time series analysis using segmented regression of the percent of pediatric patients per quarter with a computer tomography scan performed within 24-h of an emergency department visit for minor head injury per quarter

**Fig. 3:**
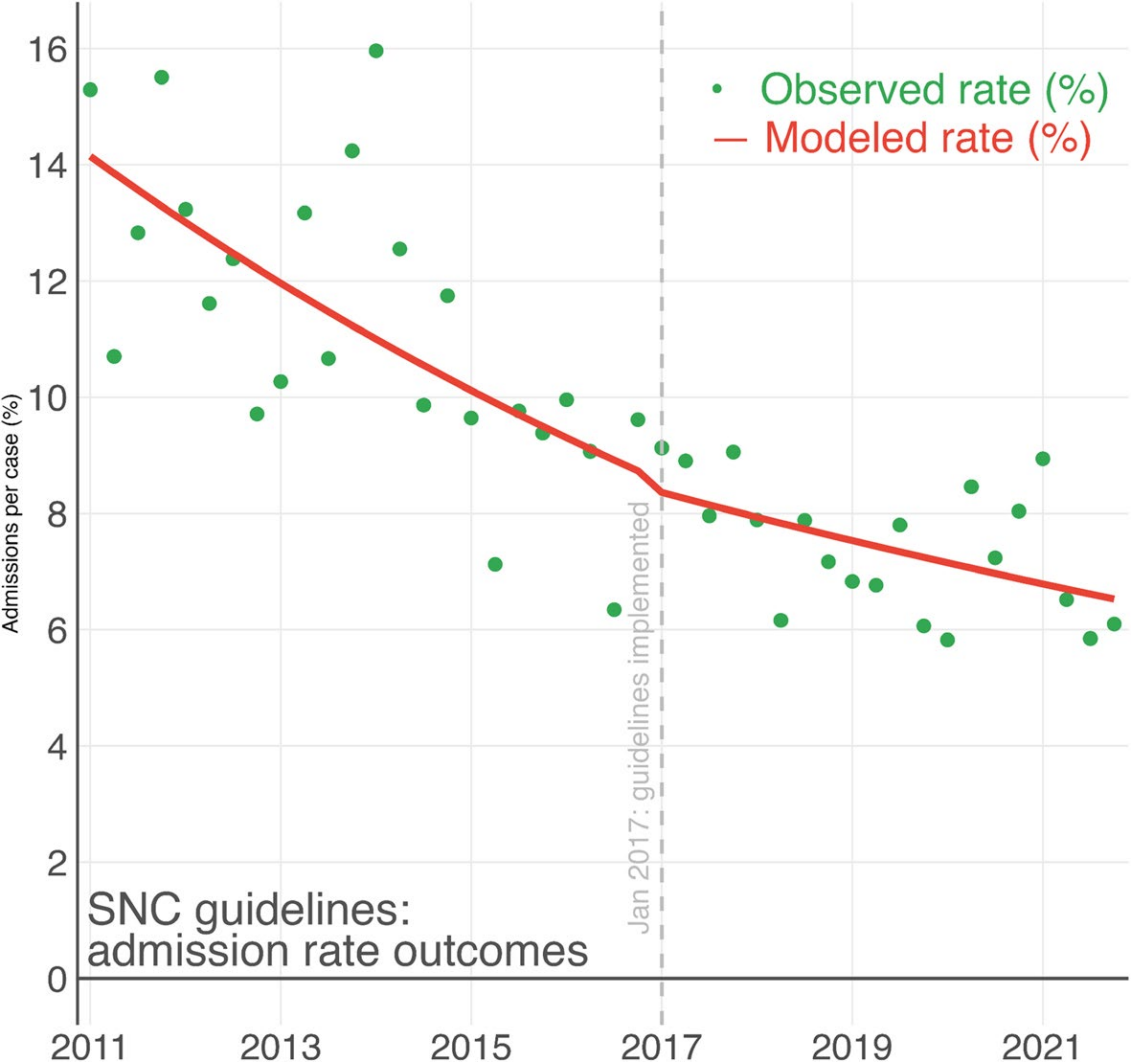
Interrupted time series analysis using segmented regression of the percent of pediatric patients per quarter being admitted after an emergency department visit for minor head injury, per quarter

In the regression time series analysis, there was a yearly decrease in proportions of patients receiving a CT scan during the pre-implementation period (14% yearly decrease) and a stationary trend (1.6% yearly decrease) during the post-implementation period (*p* < 0.001) (Fig. 2). For admissions, there is a continually decreasing trend through the whole study period with no clear post-implementation effect in the time series analysis (13% vs 12% yearly decrease, *p* = 0.6) (Fig. 3).

## Discussion

### Key results

In this retrospective observational study we observed a general decrease in CT use in pediatric ED patients with moderate and minor head injury with steady yearly decrease of 14% up until the implementation of the SNC guidelines after which the decrease flatten out to 1.6%. For admissions there was a steady decrease with no significant difference before and after the implementation of the SNC guidelines (13% vs 12% yearly decrease). The rates of serious injury and neurosurgical intervention was very low both before and after the implementation of the guidelines.

### Limitations

This study has several limitations. We could not adjudicate which patients post-implementation had been managed according to the SNC guideline and the exact effect of the SNC implementation on management is uncertain. However, the date (january 2017) when the SNC guidelines was published as a local guideline within the healthcare system is certain and there were no other interventions implemented for these patients that would explain the observed effect on CT. There were considerable differences in arrival by ambulance and triage acuity between the study periods which may affect resource utilization. However, there were no differences in adverse outcomes between the periods, and the numbers of ED attendances are similar, suggesting similar community thresholds for seeking care and disease prevalence. The differences may be related to improved risk stratification through triage, since there is considerable overlap between the SNC guidelines and the RETTS triage-guidelines for head injury in kids. Although the dataset covers all relevant outcomes within our healthcare system, we cannot exclude that patients may have been treated for relevant outcomes in other healthcare systems in Sweden. Importantly, any post-surgical or neurosurgical follow up care would be done within our health care system for patients residing within our catchment area and be captured in our dataset.

### Interpretation

While there were clear differences post and pre implementation in the number of CT scans, the time series analysis indicates that the decrease took place before the implementation of the SNC guidelines. This may be due to the awareness of low risk of adverse events and the low yield of diagnostic imaging through other guidelines, like the PECARN guidelines published in 2010 [4]. Whether the SNC guidelines prevented further decrease in imaging is uncertain but unlikely, as 2.7% by international comparison is a low rate of imaging [1].

The admissions rates were largely unaffected by the guidelines and showed a steady decrease during the whole period. These are likely driven by other factors than the guidelines, like general risk awareness in this patient group and hospital bed availability [15, 16]. Interestingly, the ED length of stay was largely unaffected by the publication of the SNC guidelines at a median of 91 and 89 min respectively. According to the SNC guidelines, minimal head injury patients may be discharged immediately and the results either suggest the guidelines had little effect on ED length of stay, likely due to length of stay primarily reflecting the waiting time prior to assessment.

In both cases of CT scans and hospital admissions, a univariate analysis would render the changes before and after implementation significant and attributable to the implementation of the SNC guidelines. The interrupted time series analysis, on the other hand, provides a more nuanced picture where reduction in CT rates happened before the guidelines were published. The change in CT rates may still be attributable to the intent of the guideline through the underlying evidence, which tends to be published before guidelines. In the case of admission rates, a univariate analysis would associate the SNC guideline implementation with a 3.6% (11% vs 7.4%) decrease in admissions based on the data in this study (Table 2). On the contrary, the time series visualizes a continuous decrease in admissions over the study period, unaffected by guideline publication, suggesting that an association is unlikely. Showing the added value of analysing implementation studies using time series analysis [12].

Our results show that the reduction in CT-imaging started, and reached a reasonable baseline, before the release of the SNC guidelines, indicating that practice changed before guideline implementation. This is likely due to the uptake of the results from clinical trials, and international decision guidelines, primarily PECARN, into practice before SNC guidelines were completed. This highlights the importance of recognising that change in practice and clinical behavior often occurs through broader dissemination of evidence and professional awareness, rather than solely through formal guideline publication. This should be considered both when evaluating the effect of guidelines and when designing guidelines for care. It also highlights the importance of evaluating trends over time to understand when change occurs, rather than relying on pre/post comparisons.

## Conclusion

There were lower rates of CT imaging and admission rates for pediatric moderate and minor head trauma after the publication of the SNC guidelines without an increased ED length of stay in this retrospective observational trial from three EDs in south east Sweden. The reduction in CT imaging was observed before the release of SNC guidelines, indicating different forms of dissemination of evidence rather than by the SNC guidelines only.

## Supplementary Information

Supplementary Material 1.

## Data Availability

The datasets used and/or analysed during the current study are available from the corresponding author on reasonable request.

## Abbreviations

CT: Computed tomography
ED: Emergency department
IQR: Interquartile Range
PECARN: Pediatric Emergency Care Applied Research Network
SNC: Scandinavian Neurotrauma Committee
SD: Standard Deviation
