# Supplementary material for: The impact of the Scandinavian Neurotrauma Committee guidelines for pediatric head trauma on the management in the emergency department—a retrospective time series analysis
Source: Scand J Trauma Resusc Emerg Med. 2026 Jan 19;34:17. doi: 10.1186/s13049-026-01554-z (PMC12849674; doi:10.1186/s13049-026-01554-z)
Supplement: Supplementary file 1 — Supplementary Material 1. [file 13049_2026_1554_MOESM1_ESM.pdf]

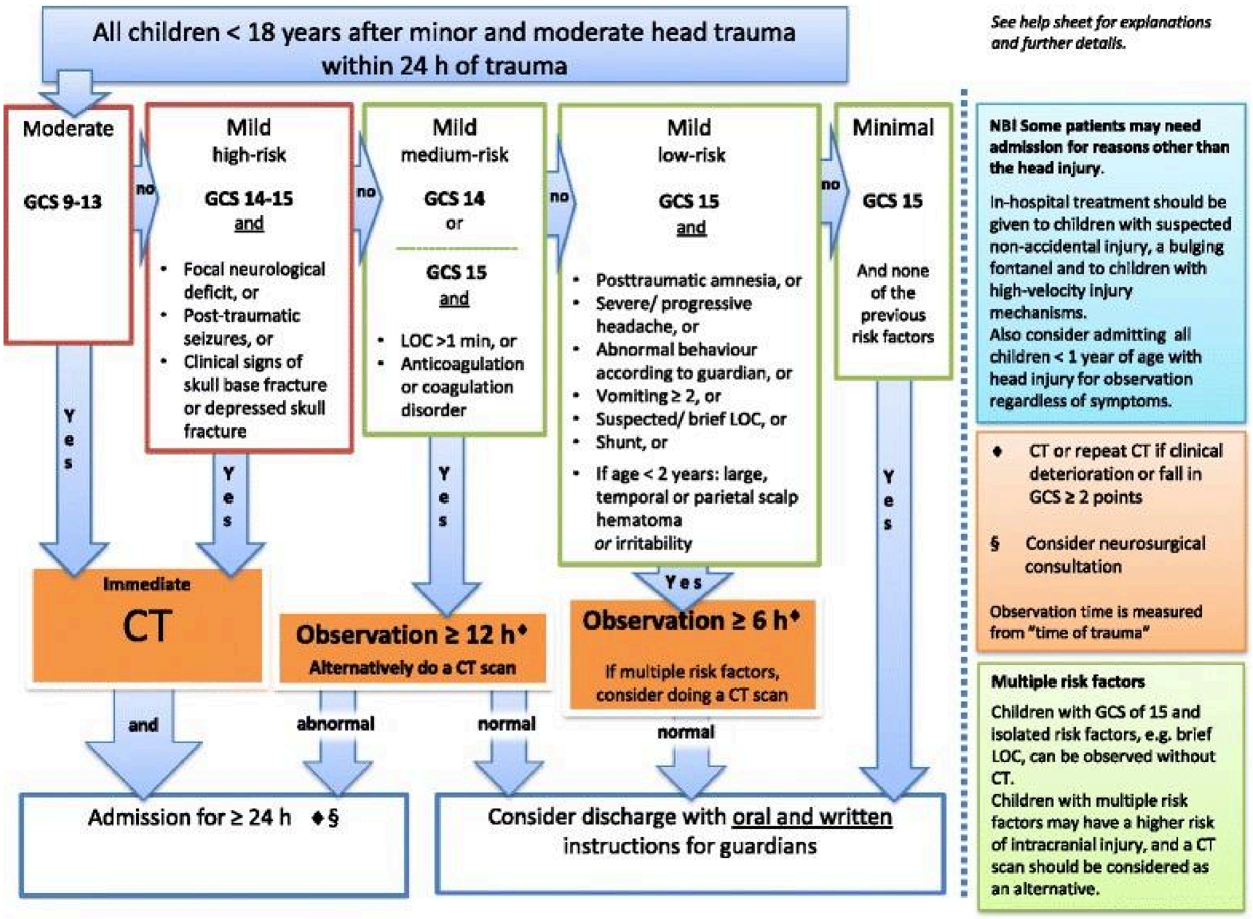

Astrand, R., Rosenlund, C., Undén, J., and Scandinavian Neurotrauma Committee (SNC), 2016. Scandinavian guidelines for initial management of minor and moderate head trauma in children. *BMC medicine*, 14 (1), 33. Republished without changes under the Creative Commons license (http://creativecommons.org/licenses/by/4.0/)
